# Effects of image distortion and Hounsfield unit variations on radiation treatment plans: An extended field-of-view reconstruction in a large bore CT scanner

**DOI:** 10.1038/s41598-020-57422-y

**Published:** 2020-01-16

**Authors:** Yong-Ki Bae, Jeong-Woo Lee, Semie Hong

**Affiliations:** 1 Department of Radiation Oncology, Konkuk University Medical Center, Seoul, Republic of Korea; 2 Department of Convergent Medical Physics, Graduate School of Engineering, Konkuk University, Seoul, Republic of Korea

**Keywords:** Breast cancer, Radiotherapy

## Abstract

This study aimed to evaluate the effect of image distortion and Hounsfield unit (HU) variation due to the extended field-of-view (eFOV) of the large-bore (LB) computed tomography (CT) on dose distribution. Both home-made inhomogeneity and breast phantoms were scanned at the geometric center position and four different offset positions. We also performed dose optimizations based on different breast phantom CT sets for evaluating the effects of image artifacts on the intensity-modulated radiation techniques. The volume changes were 0.0% to 0.5% in the air, −0.5% to 3.0% in the water, and 4.0% to 5.0% in the high-density material of the inhomogeneity phantom. Both phantoms scanning results indicate that more distortions occurred in the eFOV area due to the biased scanning center. The gamma index differences ranged from 0.87% to 4.87% for the FIF plan and from 0.52% to 6.26% for the VMAT plan. This resulted in decrease of the minimum (7.3–13.1%), maximum (−0.8–2.2%), and mean doses (−0.2–4.4%). We recommend that it should be evaluated whether the applied CT would have an appropriate eFOV range for clinical radiation treatment planning for patients.

## Introduction

In modern radiation therapy simulation, computed tomography (CT) is essential for delineating critical organs and targets, combined with other high-resolution imaging modalities, such as magnetic resonance imaging (MRI) and positron emission tomography (PET). In particular, the use of CT images enables clinicians to accommodate various electron densities of the tissues for calculating dose distributions with heterogeneity correction during dose optimization^1^. CT image calibration, which stands for electron density vs. Hounsfield Unit (HU), must be performed prior to their clinical application in the radiation treatment planning^2,3^. Generally, a large bore (LB) size allows for applying flexible set-up when a patient is scanned with sophisticated immobilization devices, such as breast tilting boards. The commercial LB models of various CT simulators provide larger bore diameters, ranging from 80 to 90 cm, than those of the diagnostic CT scanners (typically 65–70 cm)^4,5^. The LB model CTs have an extended range of HU value (−32738 to 32767) as compared to the HU range (1024 to 8191) used in the scanned images depending on the manufacturers. It also provides extended field-of-view (eFOV), larger than the scan field-of-view (sFOV) from the reconstruction algorithm^6^.

If the treated lesions are located laterally with respect to the center of the body, such as breast cancer lesions, the treatment area could be close to the edge of or outside the sFOV. In such cases, the eFOV algorithm could make image distortion and HU errors due to the partial sampling of the scanning data between the diameter of the sFOV and that of the eFOV^7^.

The image reconstruction algorithm provided by most CT scanners uses either a Filtered Back Projection (FBP) or a repetitive reconstruction method. As the FBP algorithm is a method of reconstructing scanning data within the range of the sFOV, incomplete data provided by the eFOV could result in image distortion or undesirable HU variation^8,9^.

It could lead to inaccurate and imprecise dose calculation based on the incorrectly reconstructed CT set in the treatment planning^10,11^.

In this study, we created standardized radiation treatment plans (RTPs) using tangential irradiation techniques, such as Field-in-Field (FIF) and Volumetric Modulated Arc Therapy (VMAT), based on the different CT image sets.

We analyzed the effect of image distortion and HU variation due to the eFOV on the calculated dose distribution for patients with breast cancer.

## Results

### Volume deformation and HU value variation

Table 1 shows the differences between the contoured volumes of air, water, and high-density material at the different scanned center positions, which are center, Off-20 cm, and Off-30 cm. For the reference position of scanning at the CT bore center, volume changes showed differences of 0.0–0.5% in the air, −0.5–3.0% in the water, and 4.0–5.0% in the high-density material. There were greater differences in the high-density material than in the air and water and in the Off-30 cm than in the Off-20 cm. These results indicate that the reconstructed volumes could be affected by its locations and densities in the vicinity of the range between an sFOV of 75 cm and an eFOV of 85 cm diameters. For the breast phantom, the variation of contoured volumes of the lung, heart, and left breast are listed in Table 2. Depending on the off displacements from the geometrical center, the volumes changed in the range of −0.3–12.5%, 0.1–0.5%, and 0.5–12.8% for the lung, heart, and left breast PTV, respectively. The results from both phantom scans indicate that many distortions may have occurred near the predicted eFOV boundary of Off-25 cm or 30 cm.

**Table 1:** Measured volumes in the inhomogeneity phantom. The volumes were contoured by predetermined automatic threshold values depending on the different scanning positions in the CT bore. The differences were defined as the percentage values of the difference between the center values and the offsets values.

	Air		Water		High-density material	
	Volume (cm^3^)	difference (%)	Volume (cm^3^)	difference (%)	Volume (cm^3^)	difference (%)
Center	79.7	0.0	79.6	0.0	80.1	0.0
Off-20 cm	79.7	0.0	79.2	−0.5	83.3	4.0
Off-30 cm	80.1	0.5	81.6	3.1	87.5	5.0

**Table 2:** Measured volumes in the humanoid breast phantom. The volumes were contoured by predetermined automatic threshold values depending on the different scanning positions in the CT bore. PTV, planning target volume.

	Lung		Heart		Body		Breast PTV	
	Volume (cm^3^)	difference (%)	Volume (cm^3^)	difference (%)	Volume (cm^3^)	difference (%)	Volume (cm^3^)	difference (%)
Center	462.6	0.0	164.1	0.0	1908.2	0.0	140.8	0.0
Off-15 cm	464.2	0.3	163.9	−0.1	1903.7	−0.2	140.1	0.5
Off-20 cm	469.4	1.5	163.6	−0.1	1933.6	1.3	139.7	0.8
Off-25 cm	414.5	−10.3	163.2	−0.5	1805.0	−5.4	136.4	3.1
Off-30 cm	520.5	12.5	162.4	−1.0	1917.0	0.5	122.8	12.8

**Figure 1:**
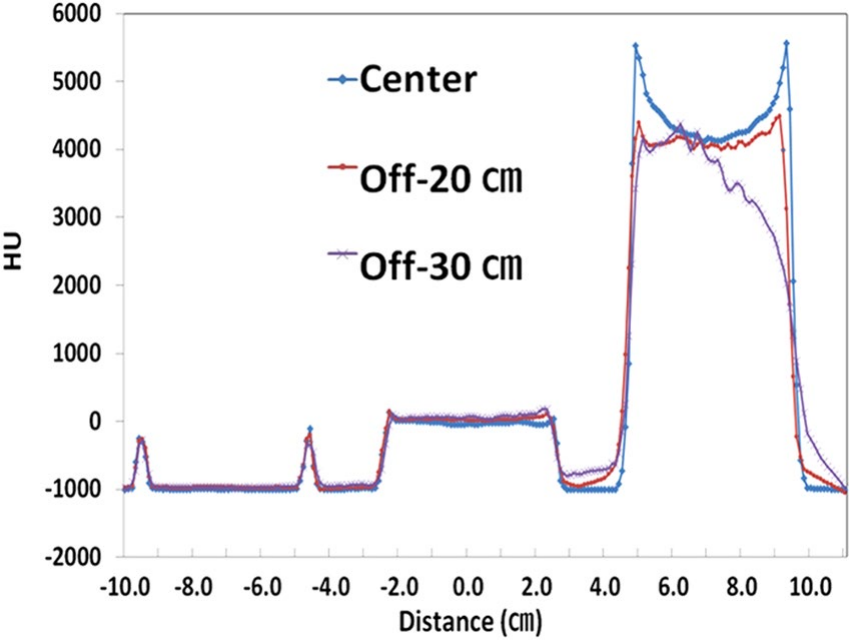
Hounsfield unit (HU) line profiles depending on the offset of scanning center using inhomogeneity phantom.

As depicted in Fig. 1, more severe variations of the HU profiles were found in the Off-30 cm CT set as compared to the center CT set, mainly in the high-density material. Table 3 shows that the HU values tended to increase in the lung and heart, while those of the left breast tended to fluctuate at the Off-25 cm and Off-30 cm. These results indicate that the HU values tend to increase with the increasing of the offset distance. However, if the offset distance is getting closer to the eFOV beyond the sFOV, the HU could be decreased.

**Table 3:** Measured mean HU values in the humanoid breast phantom depending on different scanning positions. PTV, planning target volume; HU, Hounsfield unit.

	Lung	Heart	Breast PTV
Center	−703	70	87
Off-15 cm	−694	94	82
Off-20 cm	−694	82	99
Off-25 cm	−673	96	−213
Off-30 cm	−609	115	−220

### Effects on dose distributions of breast phantom plans

**Figure 2:**
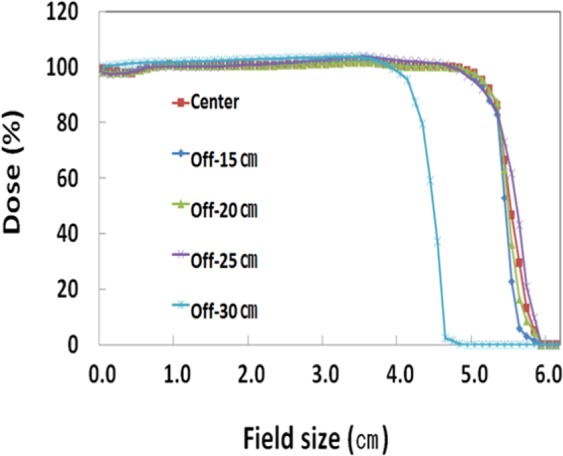
Dose line profiles in field-in-field (FIF) plans according to the different offsets of scanning centers.

**Figure 3:**
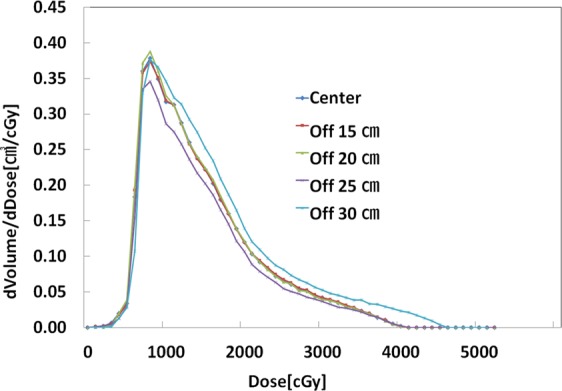
Differential dose volume histogram (DDVH) of the left lung PTV in volumetric modulated arc therapy (VMAT) plans.

**Figure 4:**
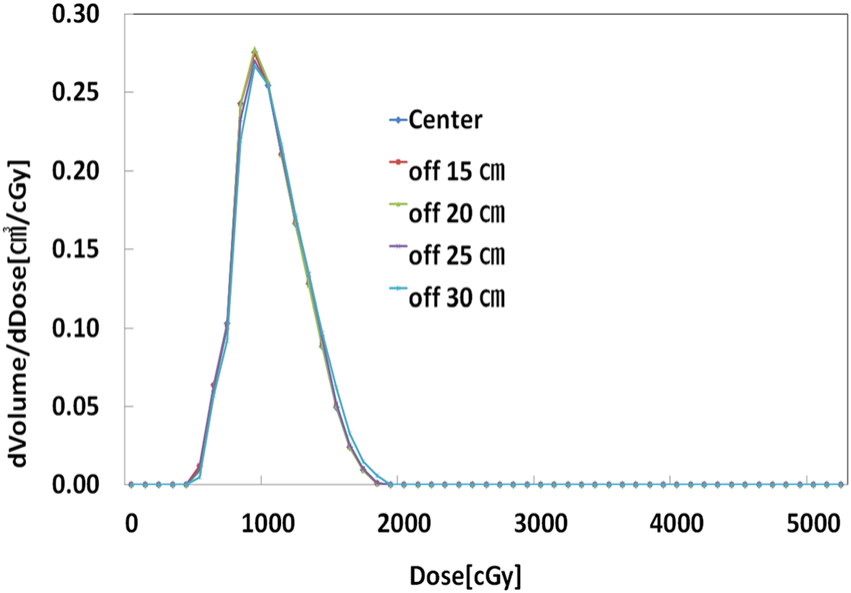
Differential dose volume histogram (DDVH) of the heart PTV in volumetric modulated arc therapy (VMAT) plans.

**Figure 5:**
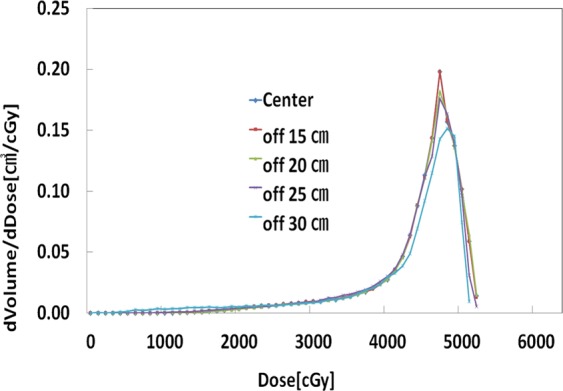
Differential dose volume histogram (DDVH) of the breast PTV in volumetric modulated arc therapy (VMAT) plans.

In the comparison of FIF plans based on the different offset-CT, the maximum doses were approximately 2% higher in the Off-25 cm and Off-30 cm than that in the center. Other than Off-30 cm, other offset plans illustrated very similar line profiles of dose distribution in the breast PTV area (Fig. 2). This finding reflects similar results from reconstruction errors of HU variations and image distortions. As shown in Table 4, in comparison to the center scanning image, GI differences ranged from 0.87 to 4.87% for the FIF plans and from 0.52 to 6.26% for the VMAT plan. The trend seen in these results also shows that the differences in GI tend to increase with increasing of the offset distances. As depicted in Figs. 3–5, the differential dose-volume histograms (DDVHs) show the effect of the image distortion on the dose distributions in the lung, heart and left breast in VMAT plans. There are many discrepancies in the range of 1000 to 5000 cGy in the DDVH of the lung (Fig. 3), DDVHs of the heart show nearly identical dose distributions (Fig. 4). A possible explanation could be the influence of the deformed lung portion in the high dose region close to the breast PTV. Table 5 and Fig. 5 show the dose variations according to the breast volume changes and a differential dose-volume comparison of breast PTV. As seen in Table 5, as offset distances increase, the volumes in the breast tend to decrease. This decrease in breast volumes reduces the minimum (7.3–13.1%), maximum (−0.8–2.2%), and mean doses (−0.2–4.4%) in the offset-CT plans.

**Table 4:** Gamma Indices % differences from FIF and VMAT plans. Gamma index criteria in this analysis were dose difference (DD) of 3% and distance to agreement (DTA) of 0.2 cm. FIF, Field-in-Field; VMAT, Volumetric Modulated Arc Therapy.

Gamma Index Difference (%)		
Types	FIF	VMAT
Off-15 cm	0.87	0.52
Off-20 cm	0.60	0.34
Off-25 cm	1.74	1.88
Off-30 cm	4.87	6.26

**Table 5:** Dose and volume statistics from cumulative dose volume histograms (CDVH) of VMAT plans. VMAT, Volumetric Modulated Arc Therapy.

	Volume (cm^3^)	Min dose (%)	Max dose (%)	Mean Dose (%)
Center	140.8	13.5	124.1	104.0
Off-15 cm	140.1	6.2	124.1	103.7
Off-20 cm	139.7	0.6	124.9	104.2
Off-25 cm	136.4	5.6	123.8	102.8
Off-30 cm	122.8	0.4	121.9	99.6

## Discussion

The use of these sophisticated positioning devices has intensified the need for CT simulators to operate with bore sizes larger than those of diagnostic CT scanners. It is critical to reconstruct accurately any portion within a geometrical CT bore range for high precision radiation treatment planning. The reconstructed CT images by eFOV were found to contain significant artifacts and image distortion, resulting in unwanted dose discrepancies during dose planning, with wide bore CT simulators (80–82 cm diameter) in many previous studies^12,13^. Wu *et al*. showed similar results regarding the dosimetric impacts of image artifacts from wide-bore CT, but there were some differences between 3D conformal radiation therapy (3D-CRT) and VMAT plans. Those results from Wu *et al*. demonstrated that the percent differences (within −0.5%) of VMAT were less than those (2–3%) of 3D-CRT plans^10^. In contrast, our study showed that VMAT plans had greater differences (0.5–6.3%) that those of 3D FIF plans (0.9–4.9%) based on breast phantom, which is very close to extended FOV limited range. These findings indicate that the dose deformation could be more influenced by the deformed breast PTV than by the HU variation. Recently, Cheung *et al*. also evaluated the impact of eFOV on the CT values and dosimetric accuracy by comparing the inserted CT calibration phantom with the mixed body phantoms. Their phantom studies demonstrated relatively small differences (less than 50 HU) for the inserts, except for the adipose, breast, and dense bone insets. Although the CT value accuracy should be within ± 20 HU from the manufacturer suggested values according to the IAEA guidelines^14^, many researchers showed that the HU variation of approximately ± 100 HU did not induce significant dose differences^15^. The artifacts caused by the inevitably biased position, such as in the breast, would result in severe volume changes that could significantly affect dose optimization, particularly in VMAT plans for breast cancer. These studies show that complex treatment techniques, such as intensity-modulated radiation therapy (IMRT), could be very vulnerable to shape and volume changes of the structures involving the dose-volume optimization^14,15^. This study has some limitations. The major limitation is that we used a home-made incomplete phantom for the planning study. For a more practical evaluation of the dose deformation due to HU variation and volume deformation close to the eFOV boundary, actual dose measurement using dedicated anthropomorphic dosimetric phantoms would be more beneficial for accurate analysis. In addition, while we found dose discrepancies around the reconstructed images close to the eFOV boundary even in the LB-CT simulator, establishing clinical guidance to avoid these errors could be difficult due to the influence of the patients’ physical conditions or the presence of additional immobilization devices in various clinical situations.

We investigated the use of the LB-CT, which has a 90-cm diameter, to evaluate the effect of the 85 cm-eFOV mode using MUSCOT algorithm with home-made inhomogeneity and humanoid breast phantoms. While image distortions did not equate to significant volume and HU variations within offset-25 cm, this offset still could be attributed to some dose distribution discrepancies in this study. The optimization of dose fluence based on segmented volumes on registered CT images for IMRT treatment could render these planning techniques more vulnerable to variations created by eFOV-based reconstructions. These variations indicate that precaution is needed when the dose calculation involves portions in the predicted eFOV area, particularly in IMRT techniques, such as VMAT and FIF. Because IMRT techniques optimize dose fluence based on segmented volumes on registered CT images, it would be more crucial to use CT images by eFOV-based reconstructions. We recommend that it should be evaluated whether the applied CT would have an appropriate range of eFOV prior to clinical application for radiation treatment planning in patients.

## Methods

**Figure 6:**
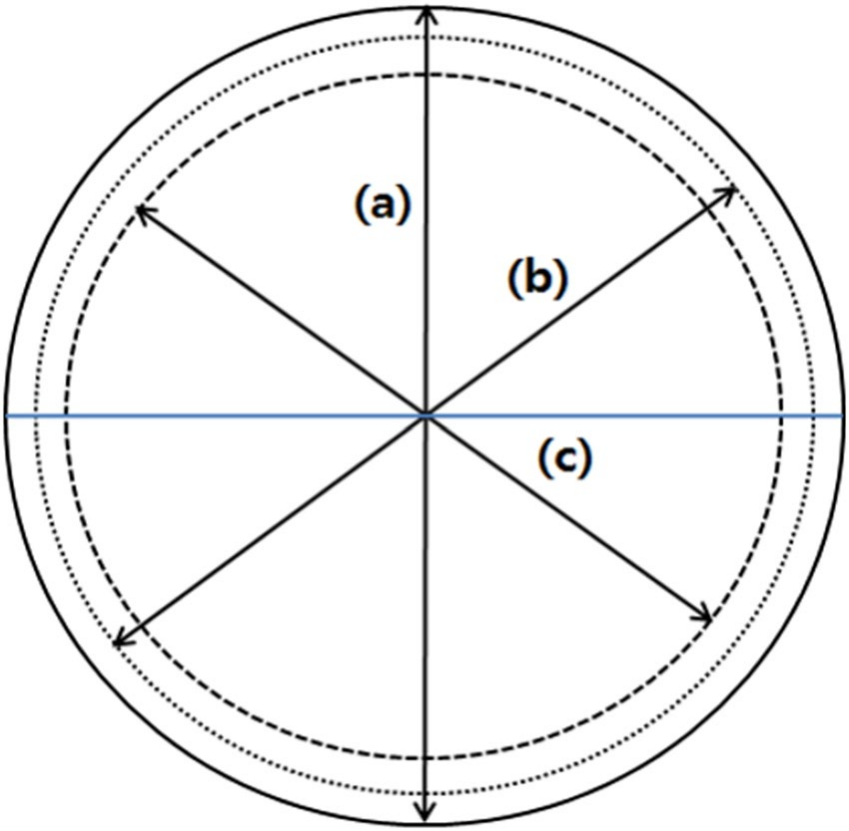
Schematic diagram of CT large bore. (**a**) Geometrical diameter (90 cm), (**b**) Extended field of view (eFOV) diameter (85 cm), and (**c**). Scanned field of view (sFOV) diameter (75 cm).

The LB CT (Aquilion LB, Toshiba, Japan) used in the experiment has a 70-cm sFOV, 85-cm eFOV, and 90-cm bore size. The scanning was performed at 120 kVp, 40 mA, 0.5 second rotation time, 300-mm scan range, and the CT images were reconstructed by 85-cm eFOV mode using multislice cone-beam tomography algorithm (MUSCOT)^16^. The inhomogeneous phantom array and a humanoid breast phantom were used in the experiments (Fig. 6).

### Two home-made phantoms for CT image acquisitions

**Figure 7:**
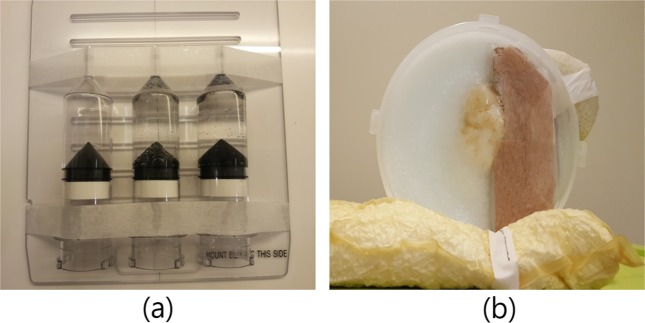
Photographs of home-made phantoms for CT scanning. (**a**) Inhomogeneity phantom with air, water, and high-density material, (**b**) Humanoid breast phantom with the lung, the heart, and the breast.

The inhomogeneous phantom array was composed of air, water, and high-density materials using 190-cm syringes (Bayer, Germany) on an acrylic plate. The high-density material was made by injection with a mixture of contrast agent (Ultravist, Bayer, Germany) and distilled water for imitating bonny density in the contrast syringes. The concentration of the injected high-density material was set to the HU value (800–1020) shown on the CT image (Fig. 7a). The home-made human phantom, which was to mimic breast and surrounding tissues, was fabricated to simulate the radiation treatment planning of breast cancer. In order to adapt the similar HU values to generic patient CT images (lung, −740 to −870; heart, 30 to 65; breast, 6 to 40) in the humanoid phantom, the lung material was chosen to cork, while the heart and breast portions were composed of thermoplastic material (Fig. 7b).

**Figure 8:**
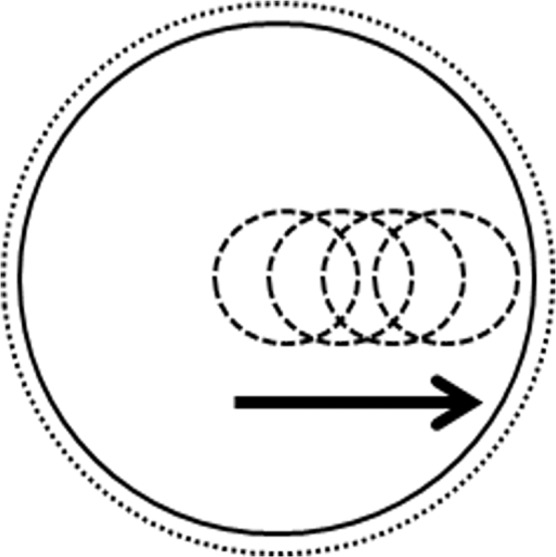
Schematic diagram of laterally offset positions of 15 cm, 20 cm, 25 cm, and 30 cm. The movement of the scanning center is from the geometrical center to offset 30 cm by 5 cm intervals.

The CT scans were performed using two phantoms with different center positions, which were at the geometrical center of the bore, and various centers shifting: Off-20 cm and Off-30 cm for the inhomogeneity array phantom and Off-15 cm, Off-20 cm, Off-25 cm, and Off-30 cm for the breast phantom (Fig. 8).

### Treatment planning in the breast phantom

The acquired CT images were transferred to the treatment planning system (Eclipse v.13.6, Varian, USA) for image registration. Based on the registered images, after organs at risk and planning target volume (PTV) delineations, the segmented volumes and HU line profiles were measured on the air, water, and high-density material of the inhomogeneous phantom array and humanoid breast phantom. The breast PTV was delineated on the breast phantom with a minus margin of 5 mm for dose-volume optimization (DVO) of VMAT. The dose prescription was 4256 cGy/16 fractions for both the FIF and VMAT plans. The dose calculation algorithm used for the FIF and VMAT planning was analytical anisotropic algorithm (AAA).

The relative priority settings for PTV, heart, and left lung for DVO were 990, 300, and 500, respectively. The applied parameters were same for the VMAT plans of the different offset CT images.

**Figure 9:**
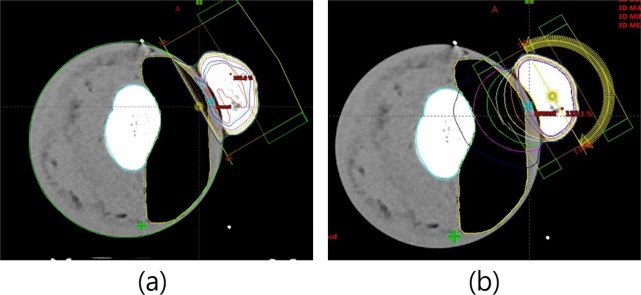
Radiation treatment planning using humanoid breast phantom. (**a**) Tangential parallel opposed pair field-in-field (FIF) plan, (**b**) 180° (330° to 150°) clock-wise volumetric modulated arc therapy (VMAT) plan.

The FIF and VMAT treatment plans were made to investigate the impact of HU variation and image distortion in the vicinity of the biased range of the reconstructed CT images through eFOV on the treatment planning results. The FIF treatment plans were made using two main tangential oblique fields with several subfields to improve dose homogeneity. The VMAT plans were also performed along the two tangentially 180° (gantry angle from 330° to 150°) clock-wise rotating volumetric modulated arc fields to cover the whole range of eFOV based on the reconstructed portion of the breast area (Fig. 9).

The dose line profile, maximum dose, and gamma index (GI) were used for analysis of the FIF plans^17^. The dose difference of 2% and distance to agreement of 2% were determined for GI calculations for analyzing the dose distributions in the FIF and VMAT plans. The commercial software for dosimetry (OmniPro-I’mrt v.1.7.0021, IBA, Germany) was used for the quantitative analysis. The DDVHs were also compared to evaluate the effect of HU variation and volume deformation of the reconstructed images on the dose distribution in breast PTV.

### Ethics approval and informed consent

Ethics approval and formal consent were not required as this was a phantom study.

## Data Availability

The datasets used and analyzed during the current study are available upon reasonable request. Please contact the authors for data requests.
